# Low-Cost Piezoelectric Sensors for Time Domain Load Monitoring of Metallic Structures During Operational and Maintenance Processes

**DOI:** 10.3390/s20051471

**Published:** 2020-03-07

**Authors:** Irene Perez-Alfaro, Daniel Gil-Hernandez, Oscar Muñoz-Navascues, Jesus Casbas-Gimenez, Juan Carlos Sanchez-Catalan, Nieves Murillo

**Affiliations:** 1 Universidad de Zaragoza, Pedro Cerbuna 12, E-50009 Zaragoza, Spain; 2 Industry and Transport Division, TECNALIA, Pº Mikeletegi 7, E-20009 Donostia-San Sebastian, Spain

**Keywords:** piezoelectric smart sensor, real-time, optimal frequency, low power, load monitoring, electromechanical impedance, μstrains, sensing, smart structures, smart manufacturing

## Abstract

The versatility of piezoelectric sensors in measurement techniques and their performance in applications has given rise to an increased interest in their use for structural and manufacturing component monitoring. They enable wireless and sensor network solutions to be developed in order to directly integrate the sensors into machines, fixtures and tools. Piezoelectric sensors increasingly compete with strain-gauges due to their wide operational temperature range, load and strain sensing accuracy, low power consumption and low cost. This research sets out the use of piezoelectric sensors for real-time monitoring of mechanical strength in metallic structures in the ongoing operational control of machinery components. The behaviour of aluminium and steel structures under flexural strength was studied using piezoelectric sensors. Variations in structural behaviour and geometry were measured, and the load and μstrains during operational conditions were quantified in the time domain at a specific frequency. The lead zirconium titanate (PZT) sensors were able to distinguish between material types and thicknesses. Moreover, this work covers frequency selection and optimisation from 20 Hz to 300 kHz. Significant differences in terms of optimal operating frequencies and sensitivity were found in both structures. The influence of the PZT voltage applied was assessed to reduce power consumption without signal loss, and calibration to μstrains and loads was performed.

## 1. Introduction

Digitalisation, the Industrial Internet of Things (IIoT) and incorporating technologies into industrial machines and processes are real necessities that are yet to be covered by Industry 4.0 [1,2]. Smart manufacturing is essential to achieve a true Industry 4.0 status in today’s production system [3], which the current advanced manufacturing industry has failed to achieve by truly adopting and integrating new and emerging solutions. Relevant cross-cutting technologies (cloud solutions, big data, analytics, sensors and communications) must also converge towards the real and dynamic monitoring of machines, processes, plants, products and goods [2]. 

Although there have been significant developments in automation and robotics, surpassing the current state of advanced manufacturing in our industry towards the smart manufacturing concept [4,5] will provide more adaptable and flexible production lines. Under this concept, multiple products could be manufactured under changing conditions, optimising resources and providing flexible solutions to increase productivity, efficiency and quality, which also lead into lower production costs [6,7]. The smart manufacturing scenario needs monitoring and digitalisation technologies need to be embedded into machine components, structures, joints and fixtures as a single element, in order to gain access to real-time information and deliver it to the IIoT network. Compiling accurate information during the operation process is conducive to the effective management of the production line and its repair and maintenance demands. Some of the future functionalities that could be provided by using heterogeneous smart sensors [8] include: early warning of ageing or damage to components, structures or elements; advanced scheduling of maintenance operations and production downtime; reduction in energy consumption; increasing visibility to reliability problems; and spare parts stock management.

The heterogeneous smart sensor concept could be defined as a combination of sensors with different capabilities (such as processing, computation and energy requirements) and functionalities (against pressure, temperature, stress, strain, chemical substances, gas, etc.). Intelligence can be added by using smart sensors, such as those based on well-known piezoelectric smart materials.

A piezoelectric material can behave like a generator when transforming mechanical stress energy into electrical energy, which is also known as the direct piezoelectric effect. Furthermore, it can work as an actuator by applying electrical force to produce mechanical deformation in the form of strain, which is also referred to as the reverse piezoelectric effect [9,10].

When a piezoelectric material is used as a sensor, the key issue is to measure admittance so that the deformation conditions of the structure can be observed based on the electromechanical coupling between the material and the sensor. In this case, the direct piezoelectric effect could be applied, not simply because of the use of lead zirconium titanate (PZT) as a generator, but in order to describe how the piezoelectric material is deformed as a result of a structural distortion [11]. An additional advantage of this type of sensor is that it exhibits low energy consumption despite being capable of producing a high excitation frequency that interrogates the structure, so it is suitable for the development of wireless sensors [12,13]. Therefore, piezoelectric sensors have been widely used in structural health monitoring [14] applications since the sensor response changes when damage appears [15,16,17,18,19,20], thereby improving safety and decreasing the maintenance costs of a wide range of civil, naval, aeronautical and transport structures. Piezoelectric sensors are also used in structural health monitoring to detect fatigue cracks, using them as an acoustic signal receiver [21,22]. In both methods to detect structural damage, either through the analysis of electromechanical impedance, or as a receiver of acoustic signals, it is a very promising technology for allowing the location of structural damage and its magnitude without destructive test utilization.

The purpose of this paper is to present the optimal frequency selection in the time domain for a piezoelectric smart sensor and its uses in strain monitoring, based on the convergence of the optimal frequency response monitoring and the real-time sensing. The identification of the optimal frequency in the time domain allows for a more accurate and stable signal of the PZT sensor compared with the methods where its identification is carried out in the frequency domain. In the present work, it is demonstrated that the characteristic frequency depends on the material nature and the geometrical magnitudes of the structure could be distinguished by different characteristic parameters of the smart sensors. Two engineering materials, aluminium and steel, were analysed under a frequency range from 20 Hz to 300 kHz and an applied load up to 6N to reach the elastic range of the materials. In addition, the influence of voltage on the susceptance response of a PZT sensor is assessed in order to achieve low power consumption responses. Likewise, the calibration of piezoelectric sensors to transform susceptibility signals into μstrains for load monitoring applications under near-flexural conditions is also presented.

### Electro-Mechanical Impedance Technique

The piezoelectric material must be in direct contact with the structure in order to use the electromechanical impedance technique for monitoring purposes. This is usually done by using an adhesive layer in a single element, called a smart structure [23]. The piezoelectric sensor is considered to have a negligible thickness (usually in the order of micrometres) and mass, compared to the structure that is to be measured. Thanks to the electromechanical coupling that occurs between the sensor and the structure, the electrical impedance of the PZT is a function of the structural parameters, so any strains will cause a change in it [24,25]. A piece of equipment known as an inductance, capacitance and resistance meter, LCR meter, is used to perform monitoring measurements, where the admittance (being the inverse of the impedance) is normally used as a direct indicator of damage [26]. 

Measuring the complex quantity of admittance, two parameters can be found; one which references the real part, conductance, and another that references the imaginary part, susceptance [24]. Although the strains or elastic or plastic deformations in the structure can be detected with both parameters, some studies reveal that, by measuring the susceptance, greater sensitivity is obtained both in the frequency domain [27,28] and in the time domain [29].

When measurements are made with a LCR meter, a harmonic voltage signal is introduced to the piezoelectric material at a specific frequency and voltage. Consequently, an excitation occurs in the sensor and it is transferred to the structure. The response of the structure to these excitations can be measured due to the electrical response that is generated in the piezoelectric material in the form of electrical conductance and susceptance; when strains are generated by applied load in the structure, the reflected signal transmits the information related to its mechanical modifications [15].

The electromechanical admittance, Y (ω), is the shared parameter between the equations of mechanical impedance of smart structures, and electrical impedance of piezoelectric sensors, as described by Equation (1) and proposed by [30].

(1) $$Y\left(\omega \right)=\omega \cdot i\cdot \frac{W\cdot l}{h}\left({\bar{\varepsilon }}_{33}^{T}\left(1-i\cdot \delta \right)-d_{31}^{2}\cdot {\bar{Y}}^{E}+\frac{Z_{a}\left(\omega \right)}{Z_{s}\left(\omega \right)+Z_{a}\left(\omega \right)}\cdot d_{31}^{2}\cdot {\bar{Y}}^{E}\cdot \left(\frac{\tan\kappa \cdot l}{\kappa \cdot l}\right)\right)$$

where W,l,h are width, length and height, the geometrical characteristics of the piezoelectric sensors are represented by the surface area, t, the thickness and d, the diameter. Its physical properties correspond to ${\bar{Y}}^{E}$, the complex Young’s modulus, ${\bar{\varepsilon }}_{33}^{T}$, the complex electric permittivity and κ, wave number. Z_s_ (ω) and Z_a_ (ω) are the mechanical and the piezoelectric impedance of the structure, respectively, where $Z_{a}\left(\omega \right)=\left(\kappa \cdot \omega \cdot t\cdot {\bar{Y}}^{E}\right)/\left(i\cdot \omega \cdot \tan\kappa \cdot d\right)$ and the wave number for one-dimensional case is $\kappa =\omega \sqrt{\rho /{\bar{Y}}^{E}}$. Equation (1) was modified by [20] to include the connection between the impedance of the structure and piezoelectric sensor as follows, Equation (2):

(2) $$Y\left(\omega \right)=\omega \cdot i\cdot \frac{d^{2}}{t}\left({\bar{\varepsilon }}_{33}^{T}-\frac{2d_{31}^{2}\cdot {\bar{Y}}^{E}}{\left(1-v\right)}+\frac{2d_{31}^{2}\cdot {\bar{Y}}^{E}}{\left(1-v\right)}\left(\frac{Z_{a,\mathrm{eff}}\left(\omega \right)}{Z_{s,\mathrm{eff}}\left(\omega \right)+Z_{a,\mathrm{eff}}\left(\omega \right)}\right)\bar{T}\right)$$

where $\bar{T}$ is the complex tangent ratio that in the ideal situation is equal to (tan κ·d)/ (κ·d). The effective impedance, Z_a,eff_ (ω) and Z_s,eff_ (ω), combines the impedance of the piezoelectric sensor and the structure; and its coupling is the reason why the impedance of the piezoelectric sensors is able to monitor the mechanical impedance of the smart structure.

## 2. Smart Sensors and Structures

### 2.1. Piezoelectric Sensors and Structural Materials

A piezoelectric sensor was adhered to a planar metallic structure to form the smart structure. Two types of material were used to develop the smart structures. The first one was aluminium 6082-T6 with a density of 2,700 Kg/m^3^, Young’s modulus of 70,000 N/mm^2^ and Poisson’s ratio of 0.33. The second one was steel S355 with a density of 7,850 Kg/m^3^, Young’s modulus of 210,000 N/mm^2^ and Poisson’s ratio of 0.3. All smart structure specimens were produced with a width of 25 and length of 325 mm. Two thickness were tested for both materials, 2 and 3 mm, to compare the influence of the thickness and the elastic nature of the material on the load behaviour of the smart structure by monitoring the impedance of the piezoelectric sensor.

The chosen piezoelectric sensor was a buzzer type PZT with reference 7BB-20-6L0 from Murata Manufacturing Co. Ltd (Kyoto, Japan). This sensor consists of a 20 mm diameter brass circumference with a thickness of 200 µm. The lead zirconate titanate material, with a diameter of 14 mm and a thickness of 220 µm, is concentric to the brass circumference; finally, a 12.8 mm diameter silver electrode is placed on top of the PZT material.

A strain gauge, CEA-06-125UN-350 from Micro-Measurements Ltd. (Raleigh, NC, USA), in the same position as the piezoelectric sensor but on the reverse side was added to all smart structure specimens in order to correlate the micro-strain measurements and to calibrate the impedance signal of the sensor.

### 2.2. Monitoring and Measurement Procedure

**Figure 1:**
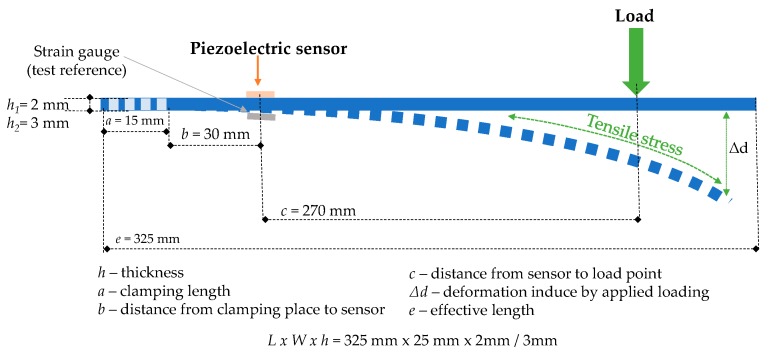
Schematic representation and characteristics dimension of the smart structure specimens.

The load measurements on the smart structures were carried out in a custom-built setup. This experimental test bench was designed to anchor one end of the planar structure while the opposite end was free to apply forces, F, in a range between 0 N and 5.9 N in six steps of around 0.98 N each in order to study the internal strain variations of each smart structure specimen by piezoelectric sensor monitoring. The load application point, the end anchor distance, the sensor and reference gauge locations, and the planar structure dimensions are represented in Figure 1. The deflection at the end of the structure (represented as Δd in Figure 1), produced by the total load applied during the tests, 5.9 N, had maximum values of 14.1 and 47.6 mm for the 3 and 2 mm thick aluminium planar structures, respectively, while the steel smart structure specimens exhibited 4.7 and 15.8 mm for the samples with 3 and 2 mm thick structures, respectively. The total deflection was three times higher in the aluminium specimen compared to the steel structures and it increased by around 336% when the specimen thickness was reduced.

The piezoelectric sensor was placed on top of the planar smart structure in order to measure the tensile stress experimented by the specimen; conversely, the strain gauge, used as a reference for micro-strain measurements, was in the compression section of the sample, as shown in Figure 1.

**Figure 2:**
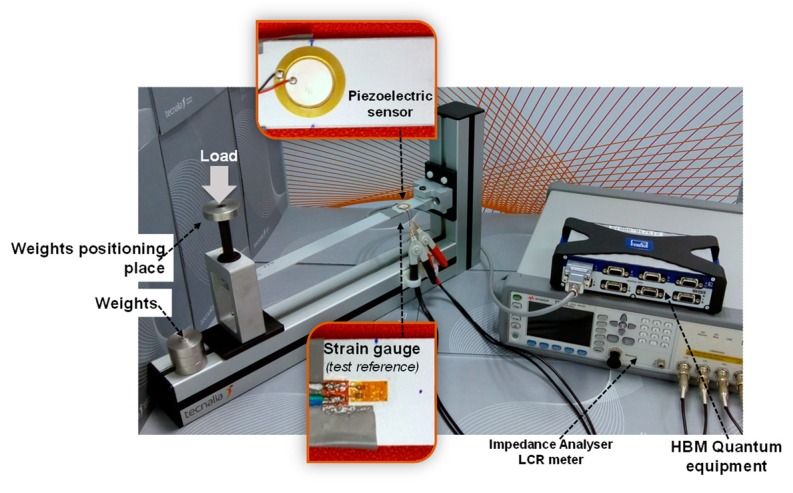
Test setup and measurement equipment.

An LCR meter, model E4980AL from Keysight Technologies, operating from 20 Hz to 300 kHz, was used to excite the piezoelectric sensor and to measure the susceptance. On the other hand, micro-strain data provided by the strain gauge were measured with the Quantum MX840B equipment from Hottinger Baldwin Messtechnik (HBM) using DC voltage, as displayed in Figure 2.

The measurements were performed in the custom-built experimental setup shown in Figure 2. The strain deformation of the sample was induced by subsequent load applications up to 5.9 N, followed by unloading to 0 N. The experiments were performed in the time domain at constant frequency and voltage. The frequency range studied was from 20 Hz to 300 kHz and two different voltages, 0.5 and 1 V, were explored for the optimal frequencies in order to analyse voltage influence.

## 3. Results. Behaviour of Smart Planar Structures with Frequency in the Time Domain

High frequencies in the range from 30 kHz to 400 kHz are often used to achieve greater sensitivity [12,18,24,31,32]. The sensitivity is contrary to the amplitude of the monitoring area [12,33]; the higher the frequency, the greater the sensitivity, but the area of the structure whose impedance is analysed is smaller, and vice versa. Therefore, a compromise must be determined with sufficient sensitivity to detect the damage, but with an area that has sufficient amplitude for the experimentation structure. However, it should be noted that prior research, using the time domain measurements at constant frequency, used lower frequencies of around 88.25 and 97.6 kHz [29]. 

In this study, the authors used the time domain measurements at constant frequency in order to monitor the strain deformations induced by the applied load in both materials, steel and aluminium, for thicknesses of *h* = 2 mm and *h* = 3 mm. The purpose was to establish a robust methodology that could be used to select the correct frequency and voltage of the piezoelectric sensor for each material and thickness. This would then allow piezoelectric sensors to be successfully calibrated to detect and quantify the strain induced by the applied load. As mentioned in [33,34], the most recent studies have shown that experiments carried out in the time domain obtain greater selectivity and sensitivity than those that use the frequency domain. Due to this fact, the authors’ first experimental step was to identify the optimal frequency for load monitoring in the smart structures. 

A frequency range of the piezoelectric sensor excitation from 20 Hz to 300 kHz was used to identify the optimal frequency with an applied load range from 0 to 3 N, instead of the nearly 6 N used in the strain calibration section of this paper. This is because the susceptance of the planar structure exhibits near-linear behaviour with the applied load for each frequency in the time domain. 

**Figure 3:**
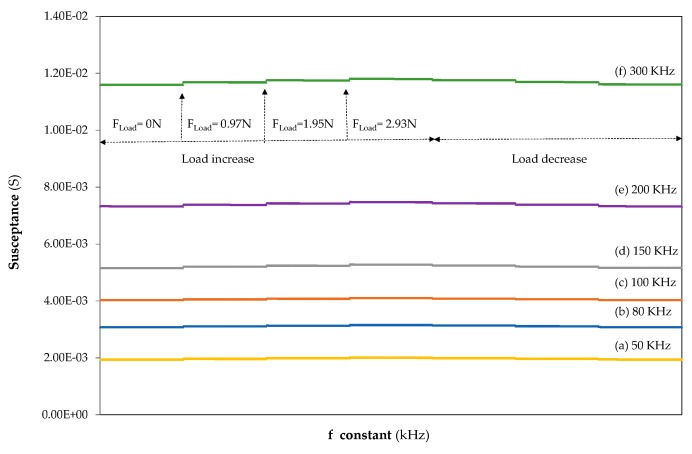
Loading and unloading variation of susceptance, absolute values at constant frequencies of (**a**) 50 kHz (yellow), (**b**) 80 kHz (blue), (**c**) 100 kHz (orange), (**d**) 150 kHz (grey), (**e**) 200 kHz (purple) and (**f**) 300 kHz (green) in the aluminium sample with *h*= 3 mm.

When a load is applied, a variation of the susceptance is observed which increases with the force acting on the material. Moreover, the susceptance increases when the frequency used during the load application is raised, as displayed in Figure 3. Both effects led to a difficult comparison and understanding of the material results, since a large number of frequencies should be explored in order to identify the most stable and optimal frequency of the piezoelectric sensor performance. In order to solve this problem, it has been proposed that the susceptance should be represented as the result of the difference between the initial susceptance, S_0,f_, at the measurement frequency without applied force, and the susceptance values, S_tn,f_, at constant frequency when incremental or decremental loads are applied. Therefore, ΔS, at the measurement frequency, was calculated as ΔS_tn,f_ = S_tn,f_−S_0,f_. 

**Figure 4:**
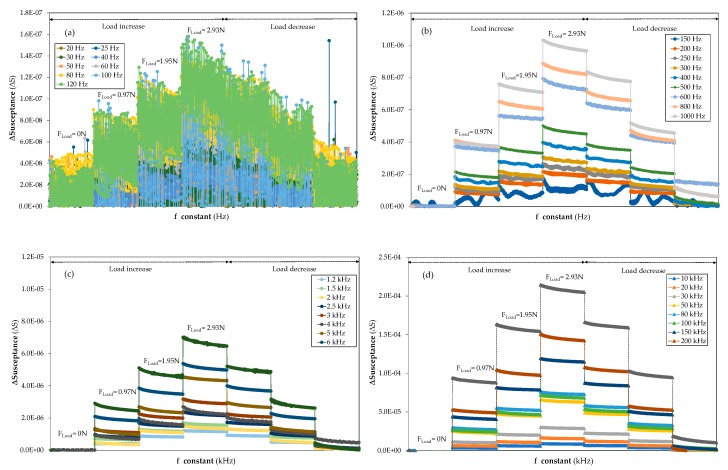
Loading and unloading effect (from F= 0N to 3N) on ΔS measured on the time domain signal at a constant frequency for a smart structure manufactured in 3 mm thick aluminium: (**a**) frequencies range from 20 to 120 Hz, (**b**) frequencies range from 150 Hz to 1 kHz, (**c**) frequencies range from 1.2 to 8 kHz and (**d**) frequencies range from 10 to 300 kHz.

The ΔS analysis of the 3 mm thick steel planar structure from 20 Hz to 300 kHz when incremental and decremental forces were applied reveals significant signal instability for lower frequencies where the influence of applied force on the susceptance could not be followed or quantified (refer to Figure 4a). This unstable behaviour of ΔS goes from 20 Hz, the initial frequency for all tests, to 120 Hz.

The ΔS becomes more stable at 150 Hz and, in general terms, a very wide frequency range was obtained from 150 Hz that allowed ΔS signals with certain stability and sensitivity to be achieved; in comparison, the frequency selection in previous studies [29] was carried out in the frequency domain, even though the loading and unloading behaviour was studied in the time domain. The improvement of the susceptance measurements in terms of stability at the different frequencies is due to the fact that the optimal frequency was identified in the time domain, leading to better results than other studies that performed the search in the frequency domain. As noted in Figure 4a, the variation of ΔS as a result of the load application is in the same range as the susceptance variation due to the natural oscillating behaviour of the piezoelectric sensor. This singularity proves that the measurement at low frequencies (up to 120 Hz) is incomplete due to the uncertainty shown by the data. Therefore, with the aim of finding stable frequencies, Figure 4b represents a frequency range from 150 Hz to 1 kHz, where it can be seen that, after 150 Hz, the different steps of increasing susceptance produced by the variation in load can already be discerned and are more stable. The ΔS behaviour at 200 Hz shows better stability than 250 and 300 Hz due to the presence of a small resonance peak. Nevertheless, the signal stability is still too poor compared with higher frequencies.

With regards to Figure 4b in particular, it is important to note that the behaviour of ΔS at 800 Hz and 1 kHz displays a small drift in the time domain measurement. This means that there is a difference, referred to as a drift, between the first and the last measured data at constant frequency, referred to as a step. These concepts shall be explained in detail in Section 4.1. As the frequency increases, the rise experienced by ΔS due to the applied load is higher than those observed at 1 kHz (refer to Figure 4c,d) so the susceptance sensitivity is still minor compared to those at higher frequencies. Similar behaviour was exhibited by the smart structure when it was exposed to the unloading process. In Figure 4c, it can be observed that, as the frequency increases, the drift is reduced and the ΔS variation when the applied load increases is also raised with the frequency. However, a little noise can be observed at 4 and 8 kHz. In Figure 4d, it can be observed that the drift trend decreases and the ΔS due to the applied load steadily increases when the frequency rises. It can also be observed that the best stability and sensitivity results were achieved in Figure 4d, compared with those at lower frequencies in Figure 4a−c. 

**Figure 5:**
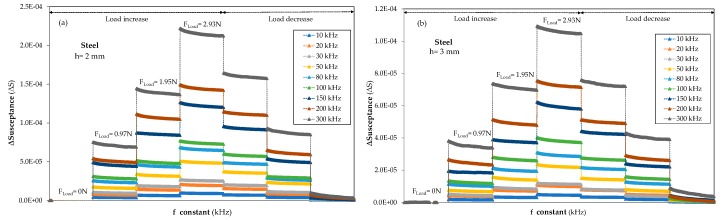

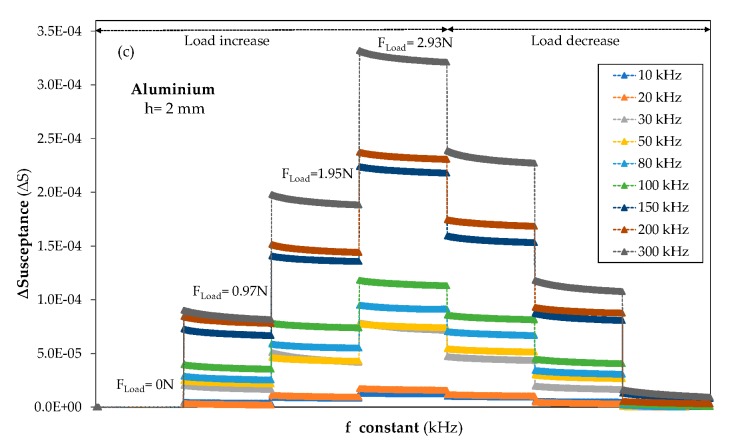
ΔS loading and unloading results (from F= 0 N to 3 N) measured on the time domain from 10 kHz to 300 kHz frequencies for (**a**) Steel specimen with *h =* 2 mm thick, (**b**) Steel sample with *h =* 3 mm thick, (**c**) Aluminium specimen with *h =* 2 mm thick.

The frequencies in the range from 10 to 300 kHz offered better stability and sensitivity in the case of the smart structure manufactured in 3 mm thick aluminium, as they also did for 2 mm and 3 mm thick steel-based smart structures (Figure 5a,b) and 2 mm thick aluminium structures (Figure 5c). In these figures, the evolution of ΔS during loading and unloading processes with loads from 0 to almost 3 N is presented in the frequency range from 10 to 300 kHz for all the measured samples. The highest values of ΔS were observed for 2 mm thick samples (Figure 5 a,c), which could be attributed to the higher level of strain induced in both materials compared with 3 mm thick samples for the same level of applied force. A similar behaviour is observed in ΔS steps for the samples with a thickness of 3 mm (Figure 4d for aluminium and Figure 5b for steel), during the increase and decrease in the applied load due to the fact that the forces applied are in the elastic behaviour range of both materials, steel and aluminium. Nevertheless, the small differences observed in the uploading steps in the case of 2 mm thick samples could be attributed to the test bench. 

As demonstrated in the results, selecting the optimum working frequency for the different materials and thicknesses in the time domain provides the greatest level of stability for the measurements. The selection of best operating frequency will be discussed in the next section.

## 4. Discussion

### 4.1. Frequency Selection

**Figure 6:**
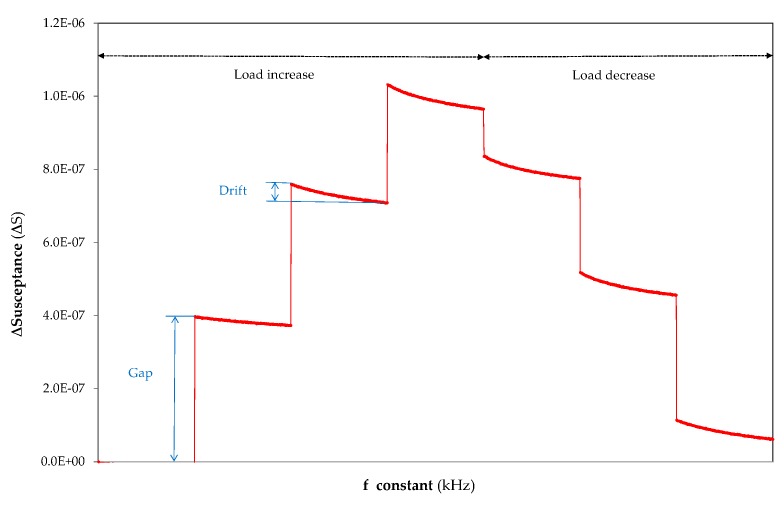
Diagram to introduce gap and drift concepts into the susceptance measurements in the time domain for the results interpretation (ΔS at 1 kHz in an aluminium sample with a thickness of 3 mm).

This section focuses on describing the methodology proposed by the authors for the optimal frequency selection when the susceptance is measured in the time domain. Firstly, two concepts should be introduced. The first concept is referred to as the gap, and it is the amplitude of the step that shows the increase in susceptance, ΔS, between two subsequent applied loads, as indicated in Figure 6. The second concept, drift, is introduced to consider signal derivation of ΔS at constant load, which is also represented in Figure 6, and it is calculated as the difference between the initial and the final values of ΔS at the constant applied force, Drift = ΔS (Initial, F = cte.) – ΔS (Final, F = cte.). The gap expresses the variation in the susceptance magnitude of the PZT sensor when the force is exerted at the load point on the planar structure. The gap reflects the sensitivity of the PZT sensor to the micro-strain induced in the sample by the force, and how subsequent applied forces influence this magnitude.

**Figure 7:**
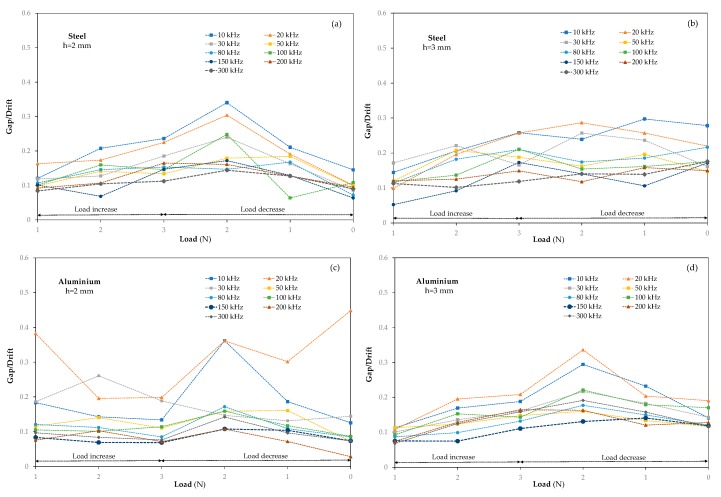
Gap/ drift ratio behaviour during load and unload test for (**a**) steel 2 mm thick, (**b**) steel 3, (**c**) aluminium 2 and (**d**) aluminium 3 mm thick.

On the other hand, the drift reflects the stability of the magnitude during a constant applied force. In order to unify the evolution of the gap during loading and unloading measurements, the ratio between gap and drift was calculated so as to counteract the influence that deviations in susceptance have on the gap. The gap and the drift were calculated by conducting loading and unloading tests on the four smart structures in the range from 0 to 3 N by applying three subsequent loads for the most stable frequency range obtained in Section 3 (from 10 kHz to 300 kHz), as displayed in Figure 7. The 2 and 3 mm thick smart steel structures, Figure 7a,b, respectively, reveal a similar behaviour for both samples, where the gap/drift ratio becomes more stable when the measurement frequency increases from 10 to 300 kHz and it decreases to its minimum value at 300 kHz. In the case of the 2 and 3 mm thick aluminium smart structures, Figure 7c,d, respectively, a similar decreasing behaviour is observed, with the difference that their minimum value occurs at 150 kHz for both aluminium specimens during loading and unloading processes. In the four samples, a significant stability was observed at the lower gap/drift ratio compared with the other frequencies. An unexpected gap/drift increase was observed during the unloading process for the first unload step in nearly all samples and regardless of the specimen thickness or material. This was most likely caused by the test bench plunger. The standard deviation, σ, of the gap/drift data has been studied in order to complete the analysis. For the resulting standard deviation values for a 3 mm thick steel specimen at f = 300 kHz, the most stable measurement observed was σ = 0.026; this is considerably lower than that obtained for the same sample at f = 150 kHz, σ = 0.048. In the case of the 2 mm thick steel smart structure, the obtained values displayed a similar tendency with σ = 0.023 at f = 300 KHz, and σ = 0.043 at f = 150 kHz. The standard deviation of gap/drift data in the aluminium samples exhibited their lowest value at f = 150 kHz in both specimens, σ = 0.028 (3 mm thick) and σ = 0.017 (2 mm thick); the values for both samples at f = 200 kHz were σ = 0.033 (3 mm thick) and σ = 0.028 (2 mm thick). The standard deviation result confirms the observations noted, that the most stable and appropriate frequency for performing time domain measurements of the susceptance in the steel smart structure is 300 kHz, while it is 150 kHz in the case of aluminium, regardless of the specimen thickness. This evidences the relationship between the frequency and the nature of the material. The results obtained seem to conclude that the best frequency to measure a structure depends on the nature of the material and that it is independent of the thickness of the structure.

### 4.2. Voltage Selection

Once the optimal frequency was selected to monitor the susceptance magnitude behaviour in the time domain under applied forces, the influence of applied voltage on the piezoelectric sensor was determined in order to identify the appropriate applied voltage for each material and thickness. 

**Figure 8:**
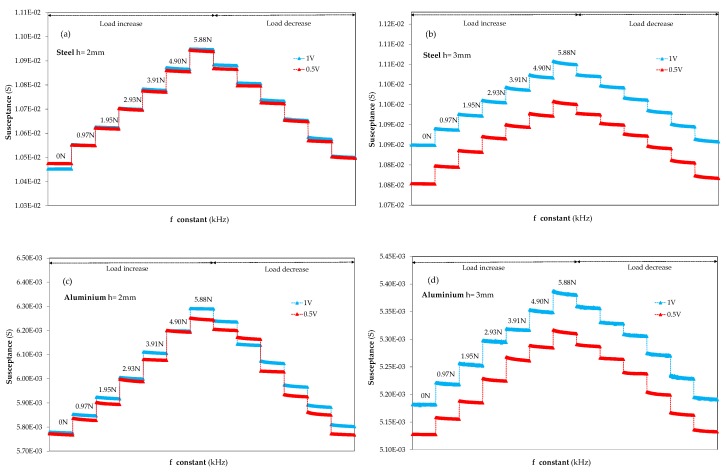
Excitation voltage influence on the time domain susceptance behavior at the optimal frequency, f optimal, steel = 300 kHz and f optimal, aluminum = 150 kHz, for (**a**) steel h = 2mm, (**b**) steel h = 3mm, (**c**) aluminum h = 2mm, (**d**) aluminum h= 3mm.

Unlike the methodology to select the optimal frequency, the range of applied forces was increased from 0 to 5.9 N in six steps for the voltage behaviour experiments. In order to optimise the applied voltage measurements, they were carried out at the optimal frequency for each material, taking into account the results of the previous section (f optimal, steel = 300 kHz and f optimal, aluminium = 150 kHz). With regards to the study on the voltage, measurements at 0.5 V were carried out to compare them with the results obtained previously corresponding to applied voltages of 1 V. On the other hand, it should be noted that in Figure 8, the susceptance behaviour at 0.5 and 1 V sensor excitation voltages was represented in the same graph for both materials and thicknesses, while the optimal frequency for each material (150 for the aluminium specimens and 300 kHz for the steel samples) was kept constant. Given that the susceptance variations due to the changes in applied voltage were lower than those caused by the changes in frequency, the absolute susceptance magnitude is shown in Figure 8 instead of ΔS, which was plotted in Figure 4 and Figure 5. 

When observing the results of the influence of applied voltage on the susceptance magnitude at the optimal frequency, and when comparing the susceptance behaviour corresponding to 3 mm thick structures for both materials (Figure 8b for steel and Figure 8d for aluminium), it can be seen that there is a visible difference between the absolute value of the susceptance magnitude at 0.5 and at 1 V for both materials.

The susceptance value increases in magnitude as the excitation voltage rises; however, its influence on the gap values due to the applied forces is near to zero because of the similarity of the steps for each load at 1 and 0.5 V. Moreover, with regards to the influence of excitation voltage on the susceptance magnitude for 2 mm thick specimens (Figure 8a in the case of steel and Figure 8c for aluminium) it was observed that the susceptance magnitude appears almost superimposed for both voltages, 0.5 and 1 V, and materials. Consequently, the well-differentiated increase exhibited in the 3 mm thick samples is not evidenced in the 2 mm thick smart structures. The influence of the sensor excitation voltage on the susceptance magnitude leads to the conclusion that the selection of the optimal excitation voltage is governed by the geometrical characteristics of the structure, in particular, by the thickness of the specimen; this contrasts with the dependency of the optimal frequency on the nature of the material demonstrated earlier. 

Moreover, other selection criteria to select the most suitable excitation voltage for susceptance measurements can also be highlighted due to the fact that there are minimal differences in the susceptance magnitude when the applied voltage of 0.5 V is doubled in 2 mm thick samples. Simultaneously, the increase in the excitation voltage to 1 V in the 3 mm thick specimens does not reflect a large increase in load sensitivity. The former results and conclusions led the authors to select 0.5 V as the excitation voltage for calibrating the smart structures. As the values of susceptance magnitude and impedance are very similar at both voltages and a decrease in the voltage does not change the impedance, using a lower excitation voltage would result in a lower energy consumption for the sensor.

### 4.3. Load Calibration of the Piezoelectric Sensors

Lastly, the piezoelectric sensor was calibrated at the optimal frequency and lower voltage in the time domain by using a conventional strain gauge to translate the susceptance magnitude variations caused by the planar structure deformation into strain units when increasing or decreasing forces are applied. 

In addition, all the measured data for each step over a period of time at the same applied force, collected for both sensors (piezoelectric and strain gauge), were mathematically converted into average values in order to compare both magnitudes, gauge μstrain and piezoelectric susceptance. 

Moreover, the reference sensor, i.e., the strain gauge, was placed on the back of the planar structure. This location means that the gauge is subjected to the reverse deformations than those suffered by the piezoelectric sensor. In order to converge both sensor signals and to convert piezoelectric susceptance into strain units, the μstrain measurements obtained from the strain gauge were analysed in its modulus form, which means no sign use at the μstrain measurements. 

**Figure 9:**
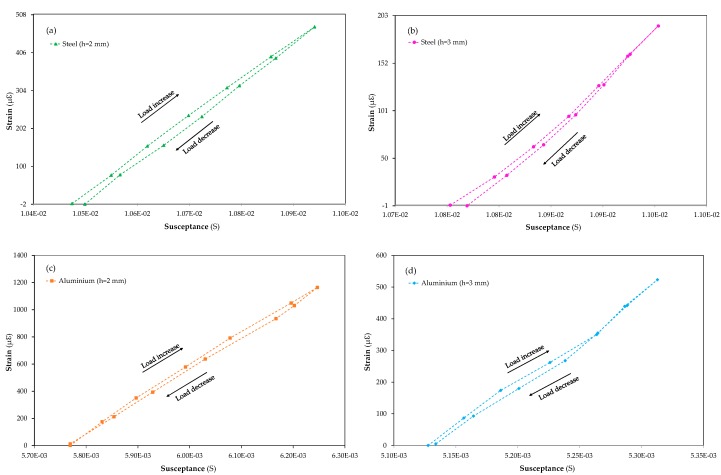
µstrain vs susceptance measurements for the calibration of (**a**) steel *h*= 2 mm, (**b**) steel *h*= 3 mm, (**c**) aluminium *h*= 2 mm and (**d**) aluminium *h*= 3 mm.

The piezoelectric sensors were calibrated under six loading and unloading steps with a maximum applied force of 5.9 N, and the measurement results for the four smart structures can be observed in Figure 9. Figure 9a,b correspond to 2 and 3 mm thick steel specimens, respectively, and Figure 9c,d correspond to the 2 and 3 mm thick aluminium samples, respectively. It can be observed in these figures that the deformations induced by the applied load led to considerably larger micro-strain values in the aluminium samples than in the steel ones. Regardless of the sample nature, a hysteresis behaviour between the loading and unloading process is observed in all the samples. In Figure 9a,b, which correspond to the steel smart structures, the last measurement of the unload step shows a value lower than zero. This fact could be assigned to the strain gauge sensor accuracy, ± 0.3%. The induced deformation in the material, aluminium, with a lower density and Young’s modulus allows a larger micro-strain to be observed compared with the steel structures. In both materials, the strain evidenced for thinner specimens is more than twice as high (1,164 μstrain and 523 μstrain in the aluminium, 2 and 3 mm thick respectively, compared with 191 μstrain and 475 μstrain in the steel, 3 and 2 mm thick respectively, for a load of 5.9 N). The ratio between steel and aluminium density or Young’s modulus is almost three, and the observed strain relation between both materials is around 2.5 at 5.9 N. 

In addition, the linear behaviour adjustment was carried out for the four smart structures in order to obtain the calibration equations to transform the time domain susceptibility measurements into deformations. The coefficient of determination, R^2^, was obtained to determine the proportion of variance in the susceptibility; it must be noted that linear settings for loading and unloading were calculated separately in order to minimise hysteresis errors. The results of these adjustments can be seen in Table 1.

The results of the four planar structures are excellent since their linear approximation is very exact, according to the R^2^ coefficient, being greater than 0.99 for all cases. Less susceptibility variance was obtained in Figure 9a,c which represent the 2 mm thick materials. 

These data suggest that the results could have a direct relationship with the thickness, because the strain gauge only has a superficial view of the deformations that the structure undergoes as a result of the measurement principle. By contrast, the piezoelectric sensor is capable of monitoring the entire volume of the surrounding area of the structure because of its electromechanical coupling with the material.

**Figure 10:**
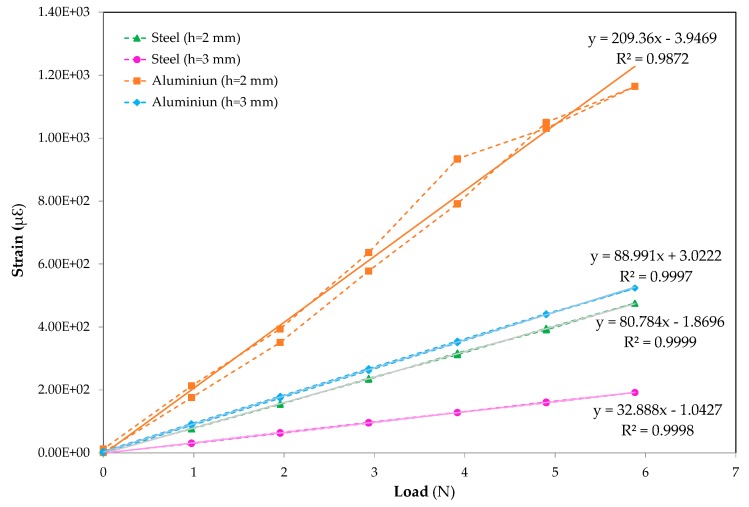
Deformation increment in micro-strains versus applied loads: ▲ steel h = 2 mm, ● steel h = 3mm, ■ aluminium h = 2 mm and ◆ aluminium h = 3 mm smart structures.

The deformation equations in μstrain for the applied forces measured by the gauge using a piece of Quantum MX840B equipment during the loading and unloading processes are displayed in Figure 10. It can be observed that the 3 mm thick aluminium exhibits similar behaviour to the 3 mm thick steel. A linear regression has been applied to each material to obtain the deformation equations, where linear behaviour is observed with a coefficient of determination near one for three of the measured specimens and there is a discrepancy in the fourth specimen due to the misalignment of the measurement at 4 N.

The following information is obtained by analysing the linear adjustments in Figure 10 and comparing the same thicknesses of different materials: (a) for 2 mm thick structures, a coefficient of 2.59 is obtained and (b) for 3 mm thick structures, a coefficient of 2.74 is obtained.

Taking into account the expression that relates the force that a structure undergoes (σ) with the strains (ε) and the material’s Young’s modulus (E), shown in Equation (3):

(3) σ=ε×E

It can be deduced that the aluminium structures have coefficients of between 2.59 and 2.74 greater than steel ones for the same thickness. Furthermore, knowing that the force applied to the different materials is the same, the Young’s modulus of steel must be greater than that of aluminium which obtained a similar coefficient. As noted earlier, the steel Young’s modulus for steel is 210 kN/mm^2^ and that of aluminium is 70 kN/mm^2^, therefore, that of steel is three times higher than that of aluminium.

**Figure 11:**
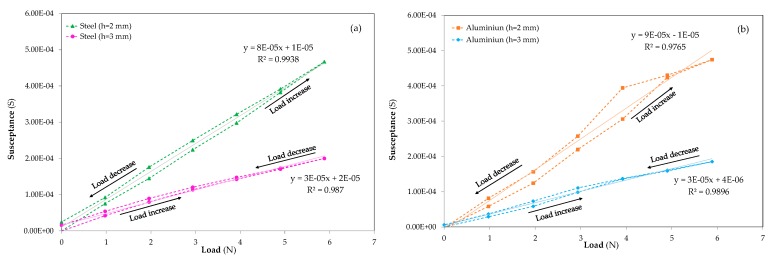
Susceptance evolution versus applied force: (**a**) steel smart structures where (▲) corresponds to h = 2 mm and (●) shows the h = 3 mm specimen results and (**b**) aluminium smart structures where (■) represents h = 2 mm sample behavior and (◆) the h = 3 mm evolution.

Finally, the increment in susceptance, ΔS, the variation obtained from the piezoelectric sensor at constant frequency in the time domain, is represented in Figure 11. As was done for incremental deformations, a linear regression has been carried out in both materials for the different thicknesses. The evolution of ΔS for steel specimens measured at a constant frequency of f = 300 kHz is represented in Figure 11a. The behaviour of the incremental susceptance for aluminium samples tested at a constant frequency of f =150 kHz is displayed in Figure 11b. Similar results to the linear calibration curves (Figure 9), and deformation evolution (Figure 10), were obtained for the coefficient of determination. Furthermore, the characteristic hysteresis of the piezoelectric sensors during loading and unloading processes is observed. As in Figure 11, the aluminium sample with h = 2 mm around 4 N exhibits an anomalous increase during the loading process that is not representative of the normal specimen behaviour and it could be assigned to a misalignment event. 

As expected, a larger increase in ΔS is observed in both materials, steel and aluminium, when the thickness of the sample decreases and the susceptance exhibits a linear increase with the applied force for both materials and thicknesses. A similar ΔS is observed in both materials, even if the susceptance measurements in the time domain were carried out under different frequencies. This fact confirms that the characteristic frequency is a property that depends on the nature of the material and the susceptance depends on geometrical properties based on the electromechanical coupling equations. In addition, although the tests were carried out at different frequencies, the similarities between the susceptance results led the authors to consider that the frequency selected for the excitation of the sensor during measurements for each material is optimal.

## 5. Conclusions

Recent developments in current engineering applications have been successful in their search for advancements towards Industry 4.0. Such breakthroughs include techniques such as smart monitoring, big data, predictive maintenance and artificial intelligence, all aimed at creating a smarter industry. These new techniques need to rely on the development of new smart sensors, and their specific electronic hardware, which are gaining ground compared to conventional ones due to their low cost and low power consumption compared with conventional strain gauges.

Advanced manufacturing processes for large components, such as aerospace, wind turbines, railway and tubing components, and, specifically, the development of more adaptable and flexible production lines where real-time information is required to increase the manufacturing efficiency, are all fields in which new smart monitoring techniques are transcendental. Furthermore, knowledge on the safety status and load history of structures, joints and fixing elements is vital. It is at this point that research into new applications for sensors capable of providing higher level information on the conditions of complex structures appears; in this field, smart piezoelectric sensors are especially valued. 

Different tests have been performed in this study, providing new techniques on how to select the conditions under which measurements are to be taken. These tests were carried out by also comparing different materials and thicknesses. This has made it possible to determine how the piezoelectric sensor is capable, in the time domain, of providing information about the operational loads and deformations supported by the structure or the component, and is also able to determine differences in materials or geometries through different parameters, frequencies and applied voltages. 

The measurements were carried out by applying successive loads at different frequencies and to different materials, wherein it has been verified that the methodology to obtain the best resolution frequency is different depending on the material. On the other hand, when applied voltage variations occurred for the same material and the same frequency, it has been observed that the signal differs depending on the thickness. It has also been demonstrated by the voltage variations that the energy consumption of the sensor can be reduced without having a significant loss in resolution or sensitivity, which could enable developments for wireless applications using this sensor technology. 

The analysis carried out in this study has also contributed novelty with respect to previous studies in that it proposes a methodology to select the optimum measurement frequency by performing previous tests in the time domain and not in the frequency domain, thus providing a more stable signal and a higher resolution. Finally, it has been observed, when comparing the piezoelectric sensor and a conventional reference sensor (a strain gauge) that the responses of both sensors exhibit linear behaviour when elastic deformation occurs. In addition, it has been noted that the thicknesses of the tested structures can be distinguished using piezoelectric signals and that, when choosing the optimum measurement frequency for each material, the susceptance signal obtained is of the same order of magnitude regardless of the material used. 

Finally, the susceptance magnitude of the piezoelectric sensors was successfully calibrated over the applied forces. 

All the results obtained show that piezoelectric sensors can be included in the new concept of smart sensors that the current market demands to be developed to face the new challenges of Industry 4.0, to monitor loads and deformations suffered by structures or components in the time domain, allowing for more efficient and smarter production lines. 

## Figures and Tables

**Table 1:** Linear calibration for the data in Figure 9. The load and the unload results were fitted separately.

$\left|\bar{\mu \varepsilon }\right|=m\times \bar{S}+n;\left(R^{2}\right)$						
	Unload			Load		
Specimens	m (10^−6^)	n (10^−4^)	R^2^	m (10^−6^)	n (10^−4^)	R^2^
Steel (2 mm)	1	−1.07	0.9995	1	−1.12	0.9994
Steel (3 mm)	0.97	−1.05	0.9922	0.97	−1.14	0.9945
Aluminium (2 mm)	2	−1.39	0.9985	2	−1.37	0.9992
Aluminium (3 mm)	3	−1.40	0.9950	3	−1.40	0.9932
